# Dr. James S. Smith

**Published:** 1912-02

**Authors:** 


Dr. James S. Smith of Buffalo, died January 30, 1912, aged 79. He was a graduate of the University of Buffalo, 1862.

